# The Impact of Insecure Attachment on Emotional Dependence on a Partner: The Mediating Role of Negative Emotional Rejection

**DOI:** 10.3390/bs14100909

**Published:** 2024-10-08

**Authors:** Janire Momeñe, Ana Estévez, Mark D. Griffiths, Patricia Macía, Marta Herrero, Leticia Olave, Itziar Iruarrizaga

**Affiliations:** 1 Psychology Department, School of Health Sciences, University of Deusto, 48080 Bilbao, Spain; aestevez@deusto.es (A.E.); m.herrero@deusto.es (M.H.); 2 International Gaming Research Unit, Psychology Department, Nottingham Trent University, Nottingham NG1 4FQ, UK; mark.griffiths@ntu.ac.uk; 3 Department of Basic Psychological Processes and Their Development, University of the Basque Country (UPV/EHU), 20018 Donostia-San Sebastián, Spain; patricia.macia@ehu.eus; 4 Faculty of Health Sciences, International University of Valencia, 46002 Valencia, Spain; leticiaolave@ucm.es; 5 Department of Experimental Psychology, Cognitive Processes and Speech Therapy, Faculty of Social Work, Complutense University of Madrid, Municipality of Pozuelo de Alarcón, 28223 Madrid, Spain; iciariru@psi.ucm.es

**Keywords:** attachment, emotional dependence, emotional regulation, emotional rejection, family relations

## Abstract

Previous evidence has demonstrated a relationship between insecure attachment and the development of emotional dependence towards an individual’s partner. However, the possibility that this relationship may be indirect and mediated by individual factors such as difficulties in emotional regulation has not previously been explored. Consequently, the objectives of the present study were to analyze the (i) differences in emotional dependence on an individual’s partner and difficulties in emotional regulation capacity according to secure, preoccupied or dismissing attachment style and (ii) mediating role of difficulties in emotional regulation in the relationship between both insecure attachment styles and emotional dependence on an individual’s partner. The sample comprised 741 participants ranging in age from 18 to 30 years (M = 21.32, SD = 2.93). The mediations were tested with linear regressions with the macro PROCESS v4.0. The results showed that emotional dependence on a partner and difficulties in emotional regulation were greater among individuals who had developed a dismissing attachment compared those with secure or preoccupied attachment. Likewise, the mediation model confirmed the mediating role of difficulties in the capacity for emotional regulation in the relationship between dismissing attachment and emotional dependence, with rejection of negative or discomfort-generating emotions predominating. The findings provide preliminary evidence that rejection of negative emotional experiences may play an important role in the relationship between insecure dismissing attachment style and emotional dependence on an individual’s partner. Consequently, it is recommended that emotional dependence intervention programs include of the management of intolerance to negative emotions.

## 1. Introduction

Bowlby [1], a pioneer in attachment theory, argued that from birth, individuals build attachment bonds with their parents or primary caregivers that influences their social and emotional development and builds the foundations on which an individual’s personality will be formed. However, although attachment theory focuses primarily on early relationships, Hazan and Shaver [2] have applied attachment principles to couple’s relationships. Although attachment style is acquired in childhood, it remains relatively stable throughout life and attachment figures shift from parents to friends and partners in adolescence and adulthood [3]. Consequently, the choice of partner and the way individuals’ behave is influenced by the experiences or quality of the bond established with parents during childhood [4]. This is how attachment theory provides understanding of individual differences reflected in the way individuals act and relate to each other throughout life [5,6]. It has also been demonstrated that the perception of the quality of an individual’s relationship established with their parents and the parents’ relationship with each other, are the best predictors of adult attachment type. This is because the experiences of living with parents during childhood are internalized and transmitted to adult couple relationships [7].

Previous studies have found that individuals who established a secure attachment during childhood and who use functional and adaptive emotional regulation strategies, have a greater capacity to regulate discomfort and emotional self-control. These individuals establish more satisfactory and less conflictive relationships than individuals who established an insecure attachment [2,8]. In contrast, individuals who established an insecure attachment during childhood have been found to have greater difficulties for emotional regulation, and difficulties in recognizing and managing their own negative emotional states [9]. They also show high levels of negative emotional experience, and dissatisfaction and conflicts within established couple relationships [10]. Insecure attachment has been distinguished into two main types: dismissing and preoccupied attachment. Individuals with a dismissing attachment are characterized by remaining involved in past attachment experiences, when the individual was a child and their signals of negative affect were probably rejected by their caregiver and their thought that expressions of negative emotion will drive others away. Therefore, the child develops an avoidant strategy of minimizing emotional distress, avoiding displaying symptoms in order to maintain proximity to the caregiver or attachment figure [11,12]. Conversely, individuals with a preoccupied attachment usually adopt a strategy of intensification of affective expressions in order to provoke a more positive and reliable response from the attachment figure. Individuals characterized by a preoccupied attachment tend to exaggerate and maximize their negative emotionality as they feel that if they relax, they run the risk of losing contact with the inconsistently available attachment figure [11].

The concept of emotional regulation refers to the ability by individuals to recognize, understand and regulate or influence their own emotions or those of others. This ability is acquired through the experiences lived with attachment figures during childhood [13] and adolescence, which is the most important period for its development [14]. Moreover, attachment style has important implications for the emotional development of individuals. In each attachment style, emotional expression and the capacity for emotional regulation varies [15]. Attachment theory provides a useful framework for understanding emotional reactions to separation and breakup in couple relationships and the process of adaptation to such painful events [16]. Consequently, establishing adequate emotional regulation is important for healthy psychological development and emotional well-being [17].

The concept of attachment is also linked to emotional dependence towards the partner since previous literature has used terms such as ‘pathological attachment’ to define it. In addition, its relationship with insecure attachment originates from early affective deficiencies [18] and difficulties in the capacity for emotional regulation have been evidenced [19]. Through the partner, individuals try to recover such unmet emotional needs that originated from childhood such as lack of support, lack of self-acceptance, and low self-esteem [20]. Moreover, the partner is positioned as a provider of personal satisfaction and security. The need for affection, attention and continuous and excessive contact from the partner, anxiety about separations, intense terror of relationship breakdown [18], and the impossibility of imagining their own existence without it seals a relationship of dependence [20]. All this can lead such individuals to place the partner at the center of their life, around whom everything revolves [21,22]. It leads to soon becoming immersed in dysfunctional [23], destructive [20] and suffocating [22] partner relationships where intense negative feelings such as emotional emptiness, low mood, anxiety, and feelings of not feeling fulfilled without the partner predominate [24].

Despite the dissatisfaction or discomfort these individuals feel in the relationship, they show a great inability to break it [18]. These individuals also manifest sharp oscillations in mood that is usually in line with the state of the relationship [21]. In addition, the breakup of the relationship does not bring the expected relief, but rather greater suffering and discomfort is experienced than what was experienced when being in the relationship. Therefore, the relationship is resumed again and again, experiencing at first a temporary relief and sense of well-being that lasts only a short time, becoming trapped in a sense of loss of freedom [20].

It should be noted that the study of emotional dependence towards a partner is important because previous empirical evidence has shown that it could favor the permanence in violent relationships by making it difficult to break it [18,25]. Irrespective of the severity of the violence suffered, the worst thing by far for individuals with emotional dependence is the breakup of the relationship and the loneliness that this would entail [21]. These difficulties in breaking up the relationship and staying in it when not being adaptive could be derived from learned attitudinal relationship styles. From the perspective of attachment theory, attachment styles in childhood can be reproduced in future relationships. In this respect, intimate relationships could be particularly affected by these patterns [26]. An individual who feels neglected and suspicious during childhood, might likely develop poor self-esteem and self-concept. Consequently, developing poor emotional regulation and relationships based on similar patterns in youth or adulthood [27].

Although the relationship between insecure attachment and emotional dependence on a partner is well established, there are no previous studies that have examined whether this relationship may be mediated by difficulties in the capacity for emotional regulation. Neither if there are differences between the insecure dismissing attachment and the insecure preoccupied attachment styles. Furthermore, the specific underlying mechanisms that explain how insecure attachment relates to emotional dependence are unknown at present. Such information is important because it would establish the direction of the psychological intervention designs. As Bowlby’s theory proposes, early childhood attachment patterns provide the child with personal resources to favorably or unfavorably face different life-risks that the child may experience [26]. It is important to explore emotional regulation as one of these personal resources for facing violent or complicated relationships. More specifically, it would be interesting to examine how emotional regulation has an influence on developing emotional dependence towards partner and staying in relationships that involve conflict.

Therefore, the main objectives of the present study were to (i) explore differences in emotional dependence toward the partner and difficulties in emotional regulation as a function of secure, preoccupied or dismissing attachment style, and (ii) analyze the potential mediating role of difficulties in emotional regulation in the relationship between preoccupied or dismissing attachment and emotional dependence toward the partner. The hypotheses (H_s_) were: (i) emotional dependence toward the partner and difficulties in emotional regulation would be greater among individuals with insecure dismissing attachment styles than among individuals with secure attachment or insecure preoccupied attachment (H_1_), considering that individuals with dismissing attachment might have higher difficulties for emotional regulation, due to the early affective deficiencies that they have experienced in the past as result of childhood experiences of rejection of their negative feelings that have led to the development of avoidant behaviors for expressing emotional distress, therefore favoring the development of emotional dependence towards the intimate partner; and (ii) the relationship between both types of insecure attachment styles (dismissing and preoccupied) and emotional dependence toward the partner would be mediated by difficulties in emotional regulation (H_2_). Dysfunctional emotional regulation will act as a risk factor for individuals who have developed an insecure attachment styles during childhood to establish emotionally dependent partner relationships.

## 2. Materials and Methods

### 2.1. Participants

The sample comprised 741 participants aged 18 to 30 years (*M* = 21.32 years, *SD* = 2.93). Over three-quarters of the participants were female (77%), 22.3% were males, and 0.7% were transgender men. Their educational level was mostly university level (96%). Regarding their occupational situation, 78.5% were students, 20.6% were workers, and 0.8% were other (e.g., unemployed).

### 2.2. Instruments

Attachment. Cartes: Modèles Individuels de Relation [28,29] (*CaMir-R*). This scale evaluates past attachment experiences retrospectively and presently, as well as family dynamics. It comprises 32 items, divided into 7 subscales: security: availability and support of attachment figures assesses the perception of being and having been loved by attachment figures, as well as being able to trust and rely on them (e.g., When I was a child, I knew that I would always find comfort in my loved ones); family worry assesses excessive worry about loved ones and separation from loved ones (e.g., I am always worried about the grief I might cause my loved ones by leaving them); parental interference assesses the perception of having been overprotected during childhood and having had fears and worries about being abandoned (e.g., My parents could not help but control everything: my appearance, my school results, and even my friends); Self-sufficiency and resentment against parents assesses feelings of resentment towards parents and rejection of dependence and affective reciprocity (e.g., From my experience as a child, I have understood that we are never good enough for parents); childhood trauma assesses memories of having had unavailable, violent or neglectful parents during childhood (e.g., Threats of separation, moving to another place, or breaking family ties are part of my childhood memories); value of parental authority assesses family values of authority and hierarchy (e.g., In family life, respect for parents is very important); parental permissiveness assesses the perception of having suffered during childhood from an absence of parental limits and guidance (e.g., My parents were unable to have authority when necessary). The first dimension is associated to a secure attachment (security: availability and support of attachment figures), defined as the perception of feeling loved and understood by attachment figures, which are reliable and are available when they are needed. The second and third dimensions refer to preoccupied attachment style (family worry and parental interference), that refer to the perception of high levels of separation anxiety from loved ones and an intense worry about the attachment figures, having been overprotected in childhood and who usually feel fear of being abandoned. The fourth dimension describes the dismissing attachment pattern (self-sufficiency and resentment against parents), that describes individuals who reject feelings of dependence towards others and affective reciprocity, and have attitudes of resentment towards loved ones. The scale format is a 5-point Likert-type scale ranging from 1 (“Strongly disagree”) to 5 (“Strongly agree”). The Spanish version has presented good psychometric properties, obtaining a Cronbach’s alpha coefficient for the subscales ranging from 0.60 to 0.85. In the present study, Cronbach’s alpha coefficient for the global scale was 0.62, and the factorial structure demonstrated a good model fit (comparative fit index [CFI] = 0.99; Tucker Lewis Index [TLI] = 0.98; root mean squared error of approximation [RMSEA] = 0.033). The results of the Harman’s test showed no significant common method variance because the one-factor solution only explained 23.5% of the variance.

Difficulties in Emotion Regulation. The Difficulties in Emotion Regulation Scale (DERS; adapted to Spanish by Hervás & Jódar) [30,31] assesses difficulties in the capacity for emotional regulation through 28 items divided into five factors: lack of control assesses difficulties in maintaining control of behavior when experiencing negative emotions (e.g., When I feel bad, I have difficulty controlling my behavior); life interference assesses difficulties in concentrating and performing tasks when experiencing negative emotions (e.g., When I feel bad, it is difficult for me to focus on other things); lack of emotional attention assesses difficulties in attending to and recognizing emotions (e.g., I pay attention to how I feel); emotional confusion assesses the difficulty in knowing and being clear about the emotions being experienced (e.g., I have difficulty understanding my feelings); and emotional rejection assesses rejection reactions to negative emotional experiences or experiences that generate discomfort (e.g., When I feel bad, I am ashamed to feel that way). The response format is Likert-type with five response options ranging from 1 (“Almost never-0–10%”) to 5 (“Almost always-91–100%”). The Spanish version the scale has good psychometric properties, obtaining a Cronbach’s alpha of 0.93 [31]. In the present study, the subscales obtained adequate internal consistency (lack of control α = 0.96; emotional rejection α = 0.90; life interference α = 0.88; lack of emotional attention α = 0.74; emotional confusion α = 0.78). The factorial structure of the scale was replicated in the present study and demonstrated a good model fit (CFI = 0.99. TLI = 0.99, RMSEA = 0.073) and, based on Harman’s test, there was no-significant common method variance (37.73%).

Emotional Dependence. The Emotional Dependence Questionnaire (CDE) [32] assesses emotional dependence on the partner by means of 23 items divided into 6 scales: separation anxiety assesses the emotional expression of fear of the possible breakup of the relationship (e.g., I am worried about the idea of being abandoned by my partner); affective expression, assesses the need for constant expressions of affection from the partner (e.g., I constantly need expressions of affection from my partner); change of plans assesses the change of plans and behaviors to satisfy the partner or to spend more time with him/her (e.g., if I have plans and my partner shows up, I change them just to be with him/her); fear of loneliness assesses the fear of not having a partner relationship and feeling unwanted (e.g., I feel a strong sense of emptiness when I am alone); borderline expression assesses impulsive actions or manifestations of self-harm in the face of a possible breakup of the relationship (e.g., I have threatened to hurt myself so that my partner will not leave me); attention seeking, assesses the attention seeking by the partner to ensure their permanence in the relationship and to try to be the center of attention in their life (e.g., I do everything possible to be the center of attention in my partner’s life). The response format is Likert-type with 6 response alternatives ranging from 1 (“Completely false about me”) to 6 (“It describes me perfectly”). The overall scale has high internal consistency with a Cronbach’s alpha coefficient of 0.93 [32]. In the present study, good psychometric properties were obtained for the following scales (emotional dependence α = 0.93; separation anxiety α = 0.89; affective expression α = 0.85; change of plans α = 0.79; fear of loneliness α = 0.82; borderline expression α = 0.50; and attention seeking α = 0.62.). The confirmatory factor analysis of the scales demonstrated a good model fit (CFI = 0.98. TLI = 0.98, RMSEA = 0.079). Harman’s test indicated the absence of significant common method variance (42.67%).

### 2.3. Procedure

Participants were recruited through two channels (i.e., online and face-to-face) within a university context. The sampling procedure was limited to individuals aged 18–30 years and which limited the influence of age bias. For the online route, surveys were made available through an online platform (surveymonkey.com, accessed on 20 September 2024). Participation was promoted through different social networks and advertisements on research websites. For the face-to-face route, participants were recruited on campus at the first author’s university who responded to the survey in paper-and-pencil format. The inclusion criteria were being 18 years of age or older and having had at least one romantic partner relationship. All participants gave their informed consent by clicking on a button indicating that the study information had been read and that they agreed to participate voluntarily in the case of the online version and by ticking the corresponding box in the case of the in-person pencil-and-paper version. Participants could leave the study at any time. The study obtained ethical approval from the Deontological Commission of the Faculty of Psychology of the Complutense University of Madrid (with reference Ref. 2020/21-035). 

### 2.4. Analytical Procedure

First, the descriptive statistics and the differences by attachment style (i.e., preoccupied vs. dismissing vs. secure attachment style) relating to difficulties in emotion regulation and emotional dependence were explored. In doing so, ANOVAs were computed with the attachment style as factor and the difficulties on emotion regulation and emotional dependence as dependent variables. Pair comparisons were developed with Bonferroni correction.

The mediations were tested with linear regressions with the macro PROCESS v4.0 [33] (Hayes, 2017). Seven models were tested applying Model 4. In all seven cases, the attachment style (i.e., preoccupied vs. dismissing vs. secure attachment style) was included as independent variable and the five difficulties in emotion regulation (i.e., lack of control, life interference, lack of emotional attention, emotional confusion, and emotional rejection) were modelled as parallel mediators. The global level of emotional dependence or each of the dimensions of emotional dependence (i.e., separation anxiety, affective expression, change of plans, fear of loneliness, borderline expression, and attention seeking) were modelled as dependent variables in each of the respective seven models. Age and gender were included as control variables due to their potential confounding effect [32,34] (e.g., Arbinaga et al., 2021; Lemos & Londoño, 2006). The indirect effect of attachment on each indicator of emotional dependence was computed with 10,000 bootstrap samples to correct for estimation bias [33] and a 95% interval confidence was computed. The datasets generated during and/or analyzed during the present study are available from the corresponding author upon reasonable request.

## 3. Results

First, the descriptive statistics and the ANOVA comparisons by attachment style are shown in Table 1. Regarding the difficulties in emotional regulation, the participants significantly differed in their levels of emotional rejection and lack of attention. Participants with dismissing attachment styles showed higher levels of emotional rejection and lack of attention than participants with secure attachment style. Regarding emotional dependence, participants differed in all emotional dependence indicators except attention seeking. More specifically, dismissing attachment style was related to higher emotional dependence, separation anxiety, affective expression, change of plans, fear of loneliness than secure, and preoccupied attachments. Borderline personality was higher for the dismissing attachment style than for the secure attachment style, but there were no differences between the dismissing and preoccupied attachment styles.

**Figure 1:**
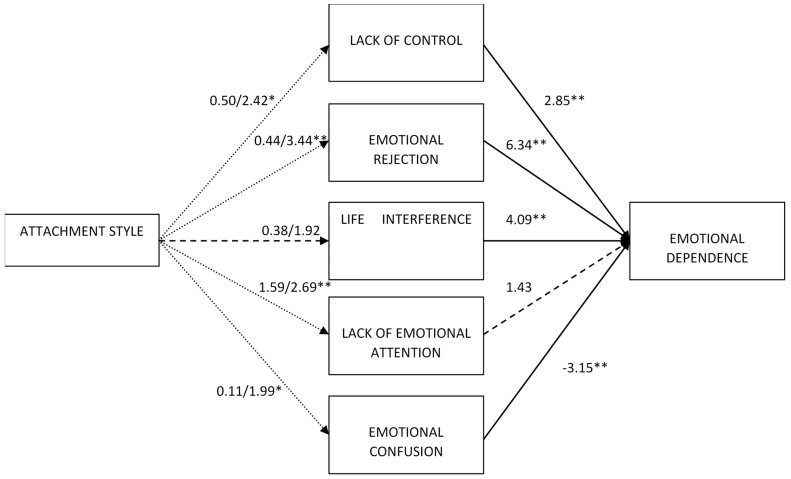
Regression effects of attachment and difficulties in emotion regulation on global emotional dependence. In the effects of attachment, the first number indicates the effect of preoccupied attachment and the second number the effect of dismissing attachment. Dotted lines indicate that the effect was only significant for dismissing attachment. Dashed lines indicate non-significant results. * *p* < 0.05, ** *p* < 0.01.

**Figure 2:**
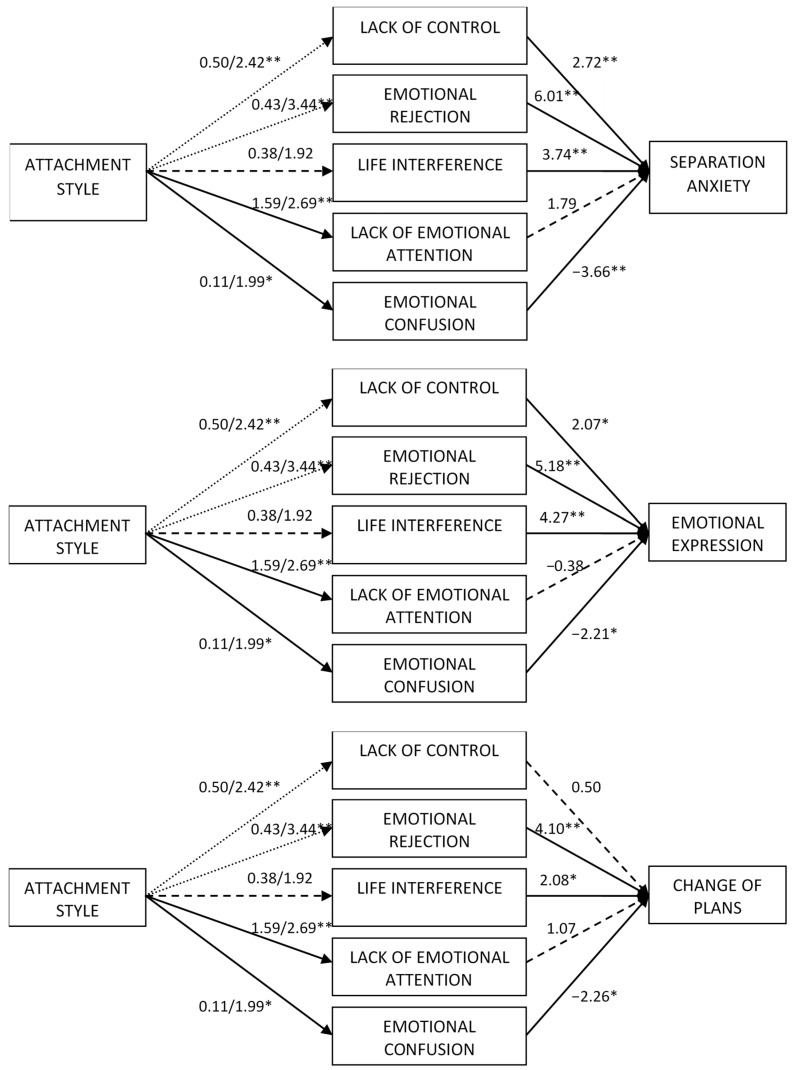
Regression effects of attachment and difficulties in emotion regulation on separation anxiety, affective expression and change of plans. Dotted lines indicate that the effect was only significant for dismissing attachment. Dashed lines indicate non-significant results. * *p* < 0.05, ** *p* < 0.01.

**Figure 3:**
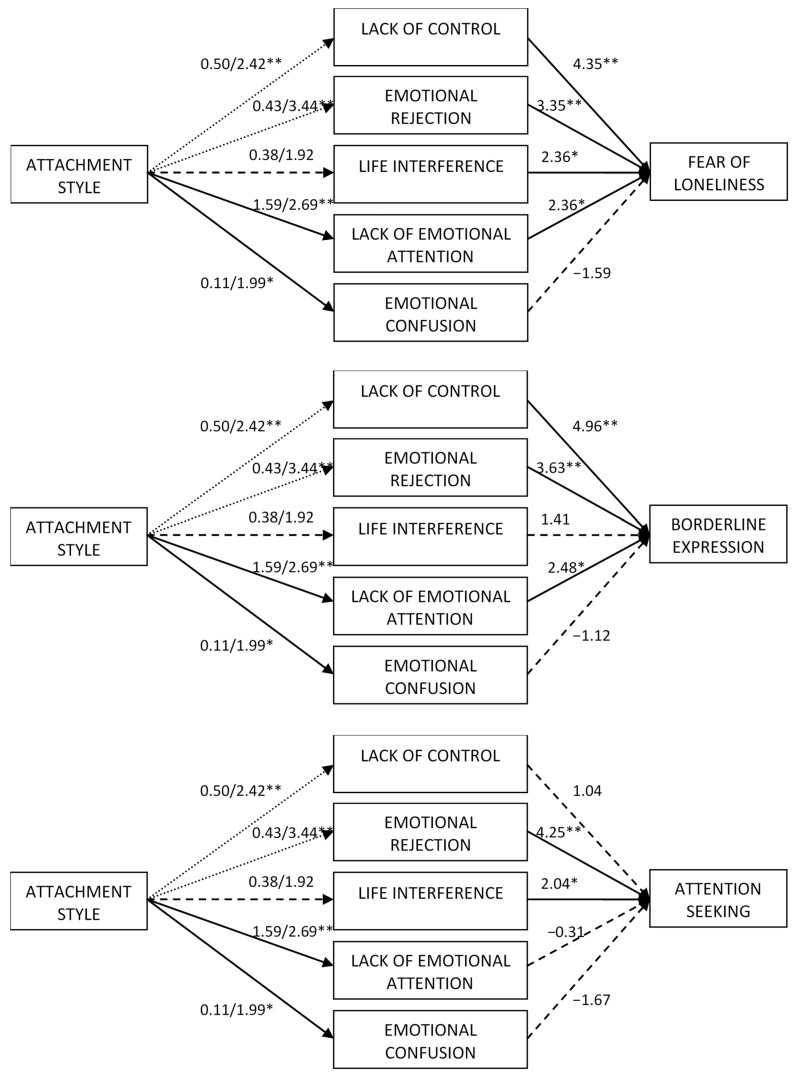
Regression effects of attachment and difficulties in emotion regulation on fear of loneliness, borderline personality and attention seeking. Dotted lines indicate that the effect was only significant for dismissing attachment. Dashed lines indicate non-significant results. * *p* < 0.05, ** *p* < 0.01.

Second, the regressions to examine the mediation effects were carried out (see Figure 1, Figure 2 and Figure 3). As observed in the *t*-test comparisons, the regression coefficients indicated that preoccupied attachment was not related to any of the emotion regulation indicators. Moreover, dismissing attachment was related to greater levels of lack of control, emotional rejection, lack of emotional attention, and emotional confusion but not to life interference (see Table 2). As shown in Table 3, when the difficulties in emotion regulation were included in the model, preoccupied attachment was not directly related to any of the indicators of emotional dependence but dismissing attachment was directly related to greater emotional dependence, separation anxiety, and borderline expression. Emotional rejection and life interference were the emotional regulation difficulties that were positively related to all indicators of emotional dependence. Lack of control was positively associated with all indicators of emotional dependence except of change of plans and attention seeking. Lack of emotional attention only had a significant positive direct effect on fear of loneliness and borderline personality. Finally, emotional confusion was negatively associated with global emotional dependence, separation anxiety, affective expression, and change of plans, but was not associated with fear of loneliness, borderline expression or attention seeking.

The indirect effects of preoccupied and dismissing attachments on the indicators of emotional dependence are shown in Table 4. As shown, none of the indirect effects of preoccupied attachment on the indicators of emotional dependence was significant. Moreover, all the mediated effects of dismissing attachment via emotional rejection were significant. Dismissing attachment, compared to secure or preoccupied attachment, was associated with higher emotional dependence by the relationship with higher emotional rejection. Emotional rejection was the only significant mediator of the effects of attachment on affective expression, change of plans, and attention seeking.

Besides emotional rejection, lack of emotional attention also significantly mediated the relationship of dismissing attachment on fear of loneliness and borderline personality. Dismissing attachment was associated with higher fear of loneliness and greater borderline expression (as opposed to secure and preoccupied attachments) due to the increment on difficulties of emotional attention.

Finally, dismissing attachment had a significant indirect effect on emotional dependence, separation anxiety, fear of loneliness, and borderline expression via lack of emotional control. Having a dismissing attachment style increased the probability of all these emotional dependence indicators by the enhancement of lack of emotional control. These models explained 26% of global emotional dependence, 22% of separation anxiety, 19% of affective expression, 13% of change of plans, 19% of fear of loneliness, 23% of borderline expression, and 13% of attention seeking.

## 4. Discussion

The present study aimed to elucidate if difficulties in emotional regulation mediated the relationship between insecure attachment and emotional dependence on the partner. The first objective of the present study was to analyze the differences in emotional dependence on the partner of participants and the difficulties in the capacity for emotional regulation as a function of the types of secure, dismissing or preoccupied attachment, established during childhood. The results confirmed the first hypothesis, showing that participants with a dismissing attachment style reported significantly higher levels of lack of control, rejection, and emotional attention than participants with secure or preoccupied attachment. These results are in line with previous studies that reported greater difficulties in the capacity for emotional regulation and avoidance patterns among individuals with an insecure dismissing attachment compared to individuals with secure attachment [35,36]. Cooke et al. [37], found that children who had developed a dismissing attachment style were less able to regulate emotions and experienced less general positive affect. Similarly, Hoover and Jackson [38], suggested that increasing the couple’s abilities to regulate their attachment-related emotions could decrease the levels of psychological aggression between the couple. Girme et al. [15] asserted that the attachment style acquired during childhood laid the foundations for emotion regulation throughout life. These authors found that individuals with an insecure attachment style tended to use emotional suppression with high frequency.

In terms of emotional dependence, individuals with dismissing attachment reported greater fear of loneliness and greater borderline expression compared to individuals with secure or preoccupied attachment. This finding is consistent with what has been previously reported in the scientific literature [18]. Similarly, Hazan and Shaver [2] noted that the characteristic of insecure attachment style among individuals with emotional dependence has its origin in the combination of traumatic experiences experienced during childhood and a perception of ambivalence in the behaviors emitted by attachment figures showing themselves sometimes warm and accessible and other times cold and distant. This inability to predict their parents’ behaviors can generate great insecurity and fear of abandonment, which leads children of such parents to a state of constant alertness and high emotional distress.

The second objective of the study was to analyze the mediating role of difficulties in emotional regulation in the relationship between the both types of insecure attachment (dismissing and preoccupied) and emotional dependence towards the partner of the participant. The results partially confirmed the hypothesized relationships between the variables. It was observed that the rejection of negative or discomfort-generating emotions was a risk factor for individuals who had acquired only a dismissing attachment style during childhood to establish emotionally dependent partner relationships. However, this was not observed in the case of the insecure preoccupied attachment style. More specifically, individuals with a dismissing attachment who employed emotional regulation strategies based on the rejection of negative emotions were more likely to report emotional dependence towards their partner. Considering previous research, insecure attachment has shown significant positive associations with emotional dependence toward the partner and difficulties in emotional regulation capacity [9]. In line with this, it has been found that individuals with a dismissing attachment style tend to predominantly experience negative emotions and employ dysfunctional strategies based on emotional inhibition [8]. Similarly, emotional dependence has been associated with difficulties in emotional regulation [20].

These results can be explained because individuals with emotional dependence tend to despise themselves [27] and present low self-esteem and negative thoughts about themselves and their personal worth [39]. Similarly, Castelló [21] notes that the dependent individual considers their partner their guide and their lifeline. This makes them feel better and allows them to avoid the intolerable feeling of loneliness and the aversion to being with themselves. It has been reported that these individuals focus all their attention and interest on their partner in order to avoid themselves and not to think about the rejection felt. Here, the purpose is to shift the focus of attention from themselves to the partner and this produces a sense of relief and attenuates their unhappiness. This leads such individuals to generate a belief that staying in a relationship with an idealized person is the solution to their pain and fear because it attenuates the excess of negative feelings that is experienced. The paradox is that a behavior that arises to reduce discomfort eventually ends up producing pain and anxiety. Moreover, Moral-Jiménez and González-Sáez [40] asserted that individuals with emotional dependence predominantly employ coping strategies based on denial and disconnection. Moreover, Muriana and Verbitz [41], point out that individuals with emotional dependence do not allow themselves to experience negative emotions such as anger, suffering or pain because the expression of these emotions could destroy or alienate the partner. Therefore, these individuals struggle for control over negative emotions and try to suppress them.

Additionally, Heshmati et al. [42] verified the mediating role of emotional suppression in the severity of grief after the breakup of a couple among individuals with insecure attachment. Likewise, previous studies have verified the mediating role of emotional regulation in the relationship between insecure attachment and other problems such as emotional eating, bulimia, depression, and emotional well-being [43,44,45]. More specifically, an insecure dismissing attachment style has been related to detrimental consequences on the physical health of individuals characterized by emotional suppression and inhibition [8].

### 4.1. Limitations

The present study is not without limitations. First, the cross-sectional design of the study cannot determine any causal relationships between the study variables, therefore the results are exploratory. Consequently, future studies should replicate and confirm the results here and observe any patterns over time using longitudinal designs. Second, it should be noted that the present study used retrospective self-report measures to assess attachment style. Therefore, social desirability might have influenced responding to the questions asked. Third, the sample predominantly comprised females and young people aged 18–30 years. The non-inclusion of individuals aged over 30 years limits the results being generalized to other older populations. Future studies could extend the heterogeneity of the sample, in order to explore if significant differences are found among older age groups.

### 4.2. Practical Implications

Despite the limitations, the results of the present study have important implications for research and clinical practice. The findings provide new evidence suggesting that a dismissing attachment style may lead to increased emotional dependence on a partner through increased use of emotional regulation strategies based on the rejection of negative or distress-generating emotions. Based on these results, among young adults with dismissing attachment, rejection of negative emotions could predominate and lead to an increase in emotional dependence on their partner. Therefore, focusing all their attention on the partner may allow them to bury or displace the focus of attention from what really causes them pain or discomfort. The dissemination of these findings is important for professionals who work in preventing future mental health problems, with the aim of conducting prevention and interventions focused on emotional expression of individuals who have a dismissing attachment style. It will be necessary to work on attachment style and interpersonal relationships, with the aim of reducing emotional dependence. This dependence could be reduced by improving emotional regulation skills, through reducing impulsivity, improving coping and resilience skills, as well as assertiveness, which would consequently promote a more secure attachment.

## 5. Conclusions

The present study highlights the harmful consequences of the rejection of negative or discomfort-generating emotions among individuals with dismissing attachment in the development of emotional dependence towards their partner. This finding is of great relevance due to the relationship between emotional dependence and the permanence in violent relationships in this period of the life cycle where first couple relationships begin to form. Data obtained in this study indicates that emotional regulation acts as a specific underlying mechanism that explains how insecure attachment is associated with emotional dependence. This information is important in order to establish the direction of the psychological intervention designs. Consequently, it could be especially useful to design prevention and intervention programs for young adults with dismissing attachment where the rejection of negative or discomfort-generating emotions is managed to reduce the establishment of dependent relationships. Consequently, future research should examine whether training in the rejection of negative emotions could be useful in decreasing the likelihood that an insecurely attached person will lead to the establishment of dependent relationships among young adults.

## Figures and Tables

**Table 1:** Descriptive Statistics and ANOVA Differences by Attachment Style.

	Descriptive Statistics								ANOVA Differences			
	Total (*n* = 741)		Secure Attachment (*n* = 667)		Preoccupied Attachment (*n* = 35)		Dismissing Attachment (*n* = 39)		Global Comparison	Secure vs. Preoccupied	Secure vs. Dismissing	Preoccupied vs. Dismissing
Variable	*M*	*SD*	*M*	*SD*	*M*	*SD*	*M*	*SD*	*F*	Δ*M*	Δ*M*	Δ*M*
Age	21.32	2.93	21.33	2.88	20.83	2.77	21.69	3.82	0.81	0.50	−0.37	−0.86
Difficulties on emotion regulation												
Lack of control	17.21	7.15	17.03	7.07	17.77	8.30	19.85	7.05	2.99	−0.74	−2.81	−2.08
Emotional rejection	14.95	6.91	14.72	6.69	15.43	7.51	18.49	8.95	5.63 **	−0.71	−3.77 **	−3.06
Life interference	10.76	4.09	10.67	4.08	11.03	4.13	11.95	4.10	1.88	−0.36	−1.28	−0.92
Lack of emotional attention	9.80	3.29	9.68	3.26	10.60	3.47	11.18	3.31	4.96 **	−0.92	−1.50 *	−0.58
Emotional confusion	9.28	3.21	9.22	3.18	9.37	3.71	10.23	3.19	1.83	−0.15	−1.01	−0.86
Emotional dependence	46.34	18.04	45.88	17.55	43.09	15.69	57.13	24.20	7.91 ***	2.79	−11.25 ***	−14.04 ***
Separation anxiety	15.02	7.25	14.82	7.07	14.11	6.31	19.12	9.59	6.89 **	0.71	−4.30 **	−5.01 **
Affective expression	10.02	4.71	9.95	4.66	9.09	4.56	11.97	5.38	4.14 *	0.87	−2.02 *	−2.89 *
Change of plans	6.60	3.17	6.56	3.11	5.89	2.43	7.97	4.16	4.66 *	0.68	−1.41 *	−2.09 *
Fear of loneliness	5.55	3.05	5.47	3.01	5.54	2.48	6.92	3.89	4.19 *	−0.07	−1.45 *	−1.38
Borderline expression	3.86	1.59	3.80	1.48	3.63	1.26	5.15	2.79	14.20 ***	0.17	−1.35 ***	1.53 ***
Attention seeking	5.28	2.27	5.27	2.26	4.83	1.99	5.97	2.53	2.54	0.44	−0.71	−1.15

Note. * *p* < 0.05; ** *p* < 0.01; *** *p* < 0.001.

**Table 2:** Regression Coefficients of the Effects of Attachment Style on Difficulties on Emotion Regulation.

	**Dependent Variable**									
	**Lack of Control**		**Emotional Rejection**		**Life Interference**		**Lack of Emotional Attention**		**Emotional Confusion**	
Independent variable	β	SE	β	SE	β	SE	β	SE	β	SE
Age	−0.30 **	0.10	−0.34 ***	0.09	−0.20 ***	0.06	−0.02	0.04	−0.17 ***	0.04
Gender										
Males	0.06	0.68	0.28	0.66	0.11	0.39	0.27	0.32	0.14	0.31
Transgender-males	4.13	3.19	−2.24	3.07	2.16	1.82	0.06	1.48	−0.10	1.43
Preoccupied attachment	0.62	1.23	0.52	1.18	0.26	0.70	0.91	0.57	0.06	0.55
Dismissing attachment	2.84 *	1.17	3.88 ***	1.13	1.29	0.67	1.46 **	0.54	1.05 *	0.53

Note. * *p* < 0.05, ** *p* < 0.01; *** *p* < 0.001.

**Table 3:** Regression Coefficients of the Effects of Attachment Style and Difficulties on Emotion Regulation on Emotional Dependence.

	Dependent Variable													
	Emotional Dependence		Separation Anxiety		Affective Expression		Change of Plans		Fear of Loneliness		Borderline Expression		Attention Seeking	
Independent variable	β	SE	β	SE	β	SE	β	SE	β	SE	β	SE	β	SE
Age	0.18	0.21	0.04	0.09	−0.01	0.06	0.10 *	0.04	<0.01	0.04	0.02	0.02	0.02	0.03
Gender														
Males	6.91 ***	1.51	2.20 ***	0.61	0.78	0.41	1.48 ***	0.29	0.82 **	0.27	0.45 ***	0.14	1.16 ***	0.21
Transgender-males	−5.96	7.09	−3.92	2.91	−1.37	1.94	−0.37	1.35	−1.16	1.25	0.64	0.64	0.23	0.97
Preoccupied attachment	−4.06	2.72	−1.24	1.12	−1.10	0.75	−0.77	0.52	−0.16	0.48	−0.28	0.25	−0.51	0.37
Dismissing attachment	5.67 *	2.63	2.28 *	1.08	0.99	0.72	0.70	0.50	0.62	0.46	0.88 ***	0.24	0.19	0.36
Difficulties on emotion regulation														
Lack of control	0.36 **	0.13	0.14 **	0.05	0.07 *	0.03	0.01	0.02	0.06 **	0.02	0.06 ***	0.01	0.02	0.02
Emotional rejection	0.73 ***	0.12	0.29 ***	0.05	0.16 ***	0.03	0.09 ***	0.02	0.09 ***	0.02	0.04 ***	0.01	0.07 ***	0.02
Life interference	0.77 ***	0.19	0.29 ***	0.08	0.22 ***	0.05	0.07 *	0.04	0.11 ***	0.03	0.02	0.02	0.05 *	0.03
Lack of emotional attention	0.28	0.20	0.14	0.08	−0.02	0.05	0.04	0.04	0.08 *	0.03	0.04 *	0.02	−0.01	0.03
Emotional confusion	−0.73 **	0.23	−0.35 ***	0.10	−0.14 *	0.06	−0.10 *	0.04	−0.06	0.04	−0.02	0.02	−0.05	0.03

Note. * *p* < 0.05, ** *p* < 0.01; *** *p* < 0.001.

**Table 4:** Indirect Effects of Preoccupied and Dismissing Attachment Styles on Emotional Dependence through Difficulties on Emotion Regulation.

	Dependent Variable						
	Emotional Dependence	Separation Anxiety	Affective Expression	Change of Plans	Fear of Loneliness	Borderline Expression	Attention Seeking
Mediator	β [95% BCI]	β [95% BCI]	β [95% BCI]	β [95% BCI]	β [95% BCI]	β [95% BCI]	β [95% BCI]
Preoccupied							
Control	0.22 [−0.80, 1.40]	0.09 [−0.31, 0.58]	0.04 [−0.16, 0.30]	0.01 [−0.09, 0.11]	0.04 [−0.13, 0.23]	0.03 [−0.11, 0.21]	0.01 [−0.06, 0.10]
Rejection	0.38 [−1.43, 2.34]	0.15 [−0.56, 0.93]	0.09 [−0.32, 0.54]	0.05 [−0.18, 0.30]	0.05 [−0.18, 0.30]	0.02 [−0.07, 0.13]	0.03 [−0.13, 0.23]
Interference	0.21 [−0.86, 1.35]	0.08 [−0.33, 0.52]	0.06 [−0.25, 0.40]	0.02 [−0.09, 0.15]	0.03 [−0.13, 0.21]	0.01 [−0.03, 0.06]	0.01 [−0.07, 0.11]
Attention	0.26 [−0.16, 0.94]	0.12 [−0.06, 0.44]	−0.02 [−0.16, 0.10]	0.04 [−0.04, 0.15]	0.07 [−0.02, 0.23]	0.04 [−0.01, 0.12]	−0.01 [−0.08, 0.05]
Confusion	−0.04 [−1.04, 0.86]	−0.02 [−0.49, 0.41]	−0.01 [−0.22, 0.17]	−0.01 [−0.15, 0.12]	<−0.01 [−0.11, 0.10]	<−0.01 [−0.05, 0.04]	<−0.01 [−0.09, 0.07]
Dismissing							
Control	1.01 [0.07, 2.41]	0.40 [0.01, 0.97]	0.20 [−0.01, 0.51]	0.03 [−0.14, 0.23]	0.17 [0.02, 0.41]	0.16 [0.03, 0.33]	0.05 [−0.05, 0.18]
Rejection	2.85 [0.72, 5.42]	1.11 [0.28, 2.13]	0.64 [0.16, 1.25]	0.35 [0.08, 0.72]	0.35 [0.08, 0.71]	0.15 [0.03, 0.31]	0.26 [0.06, 0.53]
Interference	1.00 [−0.07, 2.45]	0.37 [−0.02, 0.92]	0.28 [−0.01, 0.67]	0.10 [−0.01, 0.27]	0.14 [−0.01, 0.37]	0.03 [−0.01, 0.11]	0.07 [−0.01, 0.20]
Attention	0.41 [−0.20, 1.23]	0.21 [−0.04, 0.57]	−0.03 [−0.22, 0.15]	0.06 [−0.06, 0.20]	0.12 [0.01, 0.29]	0.06 [0.01, 0.15]	−0.01 [−0.10, 0.08]
Confusion	−0.77 [−1.85, 0.01]	−0.37 [−0.85, −0.02]	−0.15 [−0.39, 0.01]	−0.10 [−0.27, <0.01]	−0.07 [−0.21, 0.03]	−0.02 [−0.10, 0.03]	−0.06 [−0.17, 0.01]

Note. 95% BCI = 95% Bootstrap Confidence Interval; Preoccupied = Preoccupied attachment style; Dismissing = Dismissing attachment style; Control = Lack of control; Rejection = Emotional rejection; Interference = Life interference; attention = Lack of emotional attention; Confusion = Emotional confusion.

## Data Availability

The original contributions presented in the study are publicly available. This data can be found here: Figshare; https://doi.org/10.6084/m9.figshare.25933057 (accessed on 20 September 2024).
